# Identification and mechanism of wheat protein disulfide isomerase-promoted gluten network formation

**DOI:** 10.1093/pnasnexus/pgae356

**Published:** 2024-08-21

**Authors:** Jihui Gao, Jiayin Ma, Peixuan Yu, Dong Yang

**Affiliations:** Beijing Key Laboratory of Functional Food from Plant Resources, College of Food Science & Nutritional Engineering, China Agricultural University, Beijing 100083, China; Beijing Key Laboratory of Functional Food from Plant Resources, College of Food Science & Nutritional Engineering, China Agricultural University, Beijing 100083, China; Beijing Key Laboratory of Functional Food from Plant Resources, College of Food Science & Nutritional Engineering, China Agricultural University, Beijing 100083, China; Beijing Key Laboratory of Functional Food from Plant Resources, College of Food Science & Nutritional Engineering, China Agricultural University, Beijing 100083, China

**Keywords:** wheat protein disulfide isomerase, chaperone, protein folding, crosslinking, gluten network

## Abstract

Formation of the gluten network depends on glutenin crosslinking via disulfide bonds, and wheat protein disulfide isomerase (wPDI) plays an important role in this process. Here, we identify a substrate gluten protein of wPDI and the mechanism underlying wPDI-promoted glutenin crosslinking. Farinographic, rheologic, and alveographic analysis unambiguously proves that wPDI improves gluten network formation, which is directly observed by 3D reconstruction of the gluten network. Protein analysis and LC–MS/MS reveal that glutenin subunit 1Dx5 is primarily recruited by wPDI to participate in gluten network formation, and its cysteine-containing N-terminal domain (1Dx5-NTD), which harbors three cysteine residues for crosslinking, is purified. 1Dx5-NTD interacts with wPDI in both redox states, possibly folded by reduced wPDI and then catalyzed by oxidized wPDI, as further evidenced by wPDI-promoted self-crosslinking. Consistent with macroscopic observations, our results suggest that wPDI folds 1Dx5-NTD into β-strand structure that favors disulfide bond formation.

**Significance Statement**

The physical basis of dough extension, e.g. the expansion of bread during baking or the stretch of noodles, is the covalent polymerization of its molecular building block—glutenins. Currently, we know little about how this routine process takes place. Wheat protein disulfide isomerase (wPDI) is an endogenous chaperone and enzyme in wheat flour, which folds its substrate proteins and catalyzes their crosslinking. However, it is not sure how wPDI impacts dough formation. Here, we unambiguously identify that wPDI promotes the formation of glutenin network and improves dough physical properties. Subsequently, the major substrate of wPDI is identified as glutenin subunit 1Dx5. We showed that wPDI could fold the N-terminal domain of 1Dx5 into β-strand structure that helps their crosslinking.

## Introduction

As the main storage protein of wheat grains, gluten has been part of the flour-based food that endows elastic extensibility to dough for the manufacture of bread, noodles, pasta, and others since human started agriculture (1). The matrix and major component of gluten is virtually the largest protein molecule in nature, which is a glutenin polymer linked by disulfide bonds (2). Formation of new disulfide bonds between glutenins or breakage of existing bonds facilitates dough in either 3D expansion or 1D stretching (3). Protein disulfide isomerase (PDI) is a dedicated catalyst of disulfide formation reactions (4), including oxidoreductase and isomerase activity accompanied by upstream PDI oxidases (5, 6) and a chaperone that helps to fold protein intermediates that are kinetically trapped (7–9). Synthesis of glutenins in the endoplasmic reticulum and subsequent folding in the lumen is synchronized and colocalized with that of wheat protein disulfide isomerase (wPDI) (10, 11). However, no evidence has been reported on how wPDI directly interacts with glutenins and deposits them into a proteinaceous matrix in discrete bodies of wheat starchy endosperm.

Oxidative reagents, so-called dough improvers, are often needed for in vitro gluten network formation as these reagents oxidizes the cysteine residues to facilitate disulfide bond formation (12). However, the safety of azodicarbonamide, the major dough improver used in Canada and the United States, is under debate (12). Foreign and endogenous PDIs are reported able to promote gluten network formation (13, 14), and oxidants are needed for wPDI to improve bread quality (12, 15). Research has been shown that wPDI can specifically catalyze the formation of disulfide bonds which contribute positively to gluten network formation and dough rheology (16). However, others report that wPDI with oxidoreductase and isomerase activities exhibits deteriorative effects on gluten network formation, while wPDI with only chaperone activity improves the formation of gluten networks (17). Due to this contradiction in the functional mechanism of wPDI, its applications as a natural dough improver “in vitro” or molecular breeding target in vivo are hindered.

Glutenins with gliadins, which together are so-called gluten proteins, account for ∼80–85% of wheat protein (18). Unlike polymerized glutenins, gliadins occur in their monomeric form, affording dough viscosity and extensibility via electrostatic interactions and hydrogen bonding (19). According to their molecular weights, glutenins are categorized into high-molecular-weight (HMW) and low-molecular weight (LMW) glutenins (20). And HMW glutenins are highly related to the end-use quality of wheat (21). On the long arm of group 1 chromosomes of hexaploid wheat, loci *Glu1* encodes HMW glutenin subunits (GSs) with two genes linked together for the x-type and y-type HMW-GSs (22). HMW-GS allelic variation is observed on a large scale, in which 3 *Glu-1A* alleles, 11 *Glu-1B* alleles, and 7 *Glu-1D* alleles are identified, and a numbering system is developed to identify HMW-GSs (23). Among them, HWM-GS 1Dx5 exhibited a more significant effect on dough quality than any other HMW-GSs (24). In addition to the high variety in gluten proteins, it is currently unknown which protein(s) are directly recruited by wPDI into the gluten network formation.

Herein, we measure the physical properties of dough from almost every facet to unambiguously establish the role of wPDI in the formation of the gluten network. The gluten network is visualized by laser scanning confocal microscopy and 3D reconstructed. Then, quantitative analysis of the gluten network is performed to determine how gluten networks formed through wPDI. The gluten protein is analyzed and identified to find the substrate of wPDI, and their interactions in different redox states are studied. Our study illustrates the molecular basis of wPDI-promoted gluten network formation and offers a potential molecular breeding site for high-quality wheat and a candidate of natural dough improver.

## Results

### wPDI as a whole enzyme improves dough physical properties

It remains uncertain whether wPDI improves dough physical properties since results obtained with different wPDI constructs are contradictory (16, 17). Here, we employ almost all gold-standard methods to determine how wPDI addition impacts dough physical properties, including their farinographic, rheologic, and alveographic characters. To cover as much scenario of how this enzyme works as possible, dough made with wheat flours of different protein contents (grades), including low-gluten flour (LGF), medium-gluten flour (MGF), and high-gluten flour (HGF), was used in our study. The protein content in each grade of commercially purchased wheat flour was examined, and the results indicated that our experimental material covered the full spectrum of wheat protein concentrations (Fig. S1A).

**Fig. 1.**
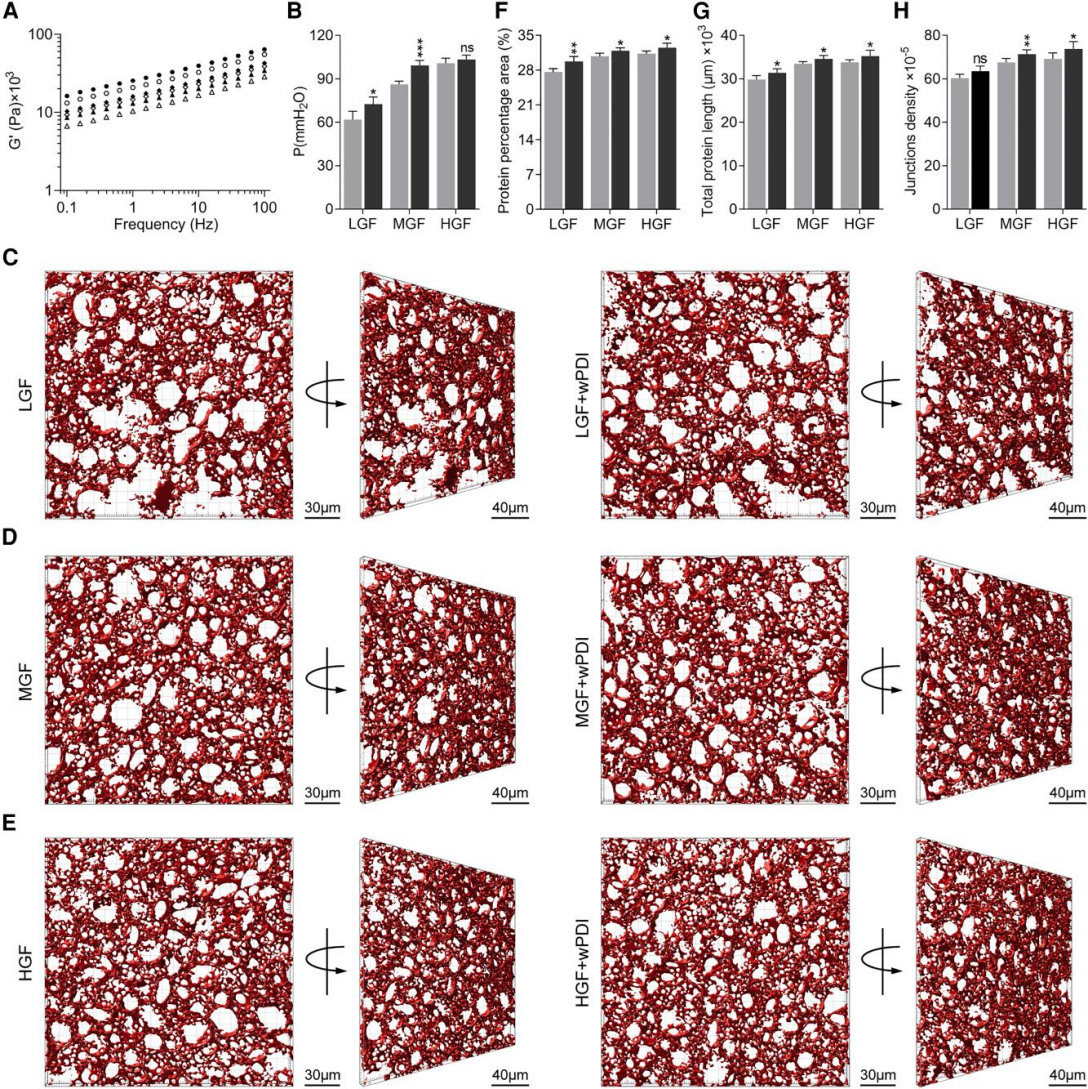
Macroproperty and microstructure changes of the gluten network induced by wPDI. A) Elastic moduli, the most representative rheologic character, of dough made with LGF (triangles), MGF (diamonds), and HGF (hexagons). Dough added with wPDI is shown in filled symbols, and dough without wPDI addition is shown in empty symbols. B) Tenacity, the most representative alveographic character, of dough made with LGF, MGF, and HGF and in the absence and presence of wPDI addition. Dough added without wPDI is shown in gray and with wPDI addition is shown in black. *** indicates *P* ≤ 0.001, ** indicates 0.001 < *P* ≤ 0.01, * indicates 0.01 < *P* ≤ 0.05, and ns stands for not significant. C–E) 3D gluten network in the absence and presence of wPDI added to dough made of LGF, MGF, and HGF. Dough with rhodamine B staining is examined with confocal laser scanning microscopy, and the 3D gluten network (red) is reconstructed. The spatial gluten network is tilted in each sample, and the empty holes are starch granules that could not be stained and visualized. Scale bars represent 30 and 40 μm, respectively. F–H) Protein percentage area, “total protein length,” and junction density by quantitatively analyzing the gluten network of each of the above samples. Dough added with wPDI is shown in gray and without wPDI is shown in black. *P* values indicate the same.

Farinographic analysis characterized the dough formation process, and dough made with different grades of flour was examined with the addition of a series of concentrations of wPDI. The dough development time generally increased as the protein content increased and further increased as more wPDI was added (Fig. S1B), suggesting a longer time of dough formation. Rheologic and alveographic analyses characterized the uniaxial and biaxial extension of dough, respectively. Dough exhibited higher G′ values when it contained higher-grade flours, and its G′ values further increased once wPDI was added (Fig. 1A), indicating that wPDI addition improved uniaxial dough elasticity. These results were in line with alveographic measurements showing that dough tenacity and baking strength increased with higher flour grade and further increased with wPDI addition (Fig. 1B and Fig. S1C). All these indicators indicated that a stronger gluten network formed in dough with higher protein content or with wPDI addition at a fixed protein content. Additionally, an improved dough viscosity was also observed in the presence of wPDI (Fig. S1D), as suggested by higher G'' values with wPDI addition.

### wPDI promotes gluten network formation

To directly observe the gluten network formed in the presence of wPDI, dough was prepared with rhodamine B to stain the gluten proteins and examined under confocal laser scanning microscopy. The scanning images were reconstructed into 3D views of “in vitro” gluten networks. Through these images, we could visualize the gluten protein (red area) knitting together with empty holes, large or small, of starch granules which could not be visualized by staining with rhodamine B (Fig. 1C–E). When corresponding 2D images of gluten networks from different samples were quantitatively analyzed, it could be seen that the protein percentage area increased as the protein content increased (Fig. 1F). Similarly, the total protein length (an indicator of total proteins participated in a gluten network) and junction density increased in dough made with higher-grade flours (Fig. 1G and H). Compared to dough without wPDI addition, dough added with wPDI further improved these characters among all the groups, directly showing that a stronger gluten network formed as a result of wPDI addition.

**Fig. 2.**
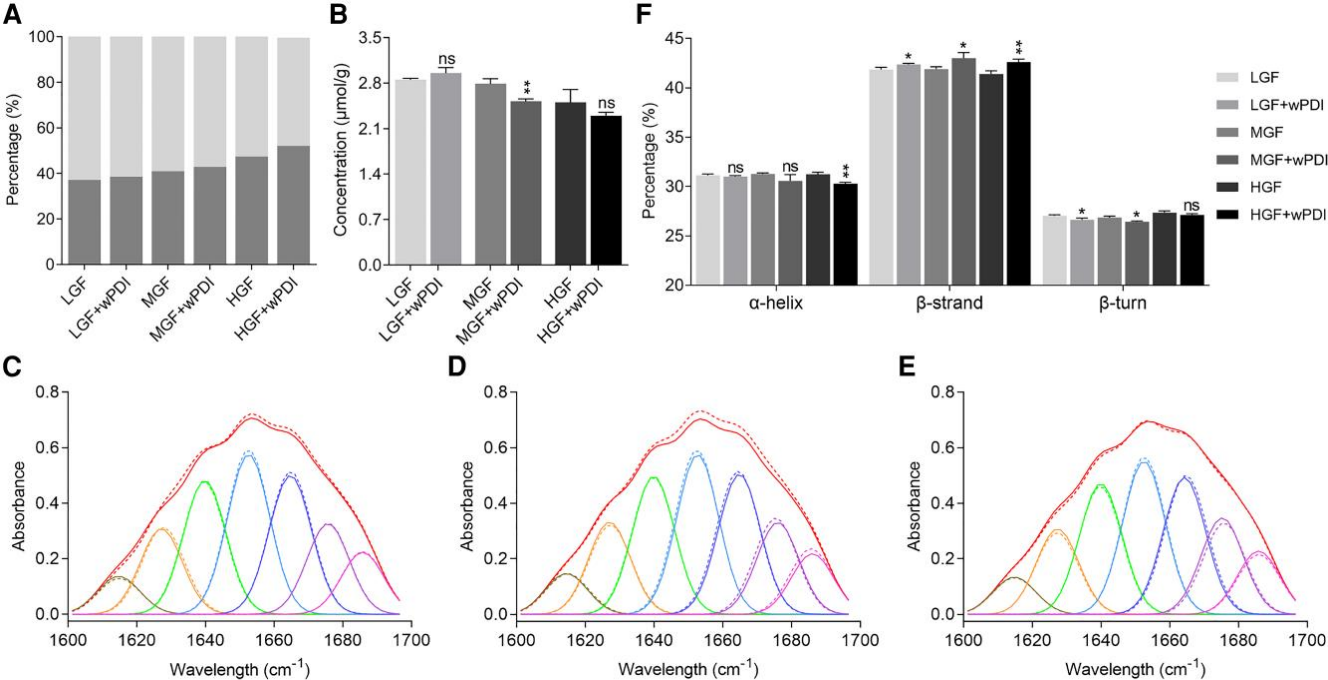
Gluten protein distribution, crosslinking, and secondary structure change induced by wPDI. A) Proportion of SDS-soluble and SDS-insoluble proteins, in which the latter is generally considered to participate in the gluten network formation, is measured in dough produced from LGF, MGF, and HGF in the absence and presence of wPDI, respectively. Light gray indicates SDS-soluble proteins, and dark gray indicates SDS-insoluble proteins. B) Free sulfhydryl contents in gluten proteins made with LGF, MGF, and HGF in the absence and presence of wPDI, respectively. *** indicates *P* ≤ 0.001, ** indicates 0.001 < *P* ≤ 0.01, * indicates 0.01 < *P* ≤ 0.05, and ns stands for not significant. C–E) FTIR second-derivative spectra and Fourier deconvoluted amide I band of the gluten network in dough made with LGF, MGF, and HGF in the absence of wPDI. The deconvoluted amide I band region (1,600–1,700 cm^−1^) is assigned to extended chains (1,600–1,615 cm^−1^), β-strands (1,624–1,640, 1,681 cm^−1^), random coils (1,640–1,650 cm^−1^), α-helices (1,650–1,660 cm^−1^), and β-turns (1,660–1,670, 1,694 cm^−1^). Dashed line indicates secondary structures of gluten proteins made with LGF, MGF, and HGF in the presence of wPDI. F) Derived proportions of secondary structures of gluten proteins made with LGF, MGF, and HGF in the absence or presence of wPDI. *P* values indicate the same.

According to their participation in the gluten network formation, these crosslinked gluten proteins are referred to as glutenin macropolymer (GMP) or SDS-insoluble proteins (25). Quantification of these proteins revealed that the GMP portion increased in dough produced with higher-grade flours and wPDI addition further increased their GMP portions (Fig. 2A), indicating that wPDI could promote protein participation in gluten network formation. The free sulfhydryl content, another indicator of gluten network formation, was measured in the above dough (26). It generally decreased in dough made with higher-grade flours, and wPDI addition further decreased the free sulfhydryl contents in the MGF and HGF groups (Fig. 2B). The total gluten protein secondary structures were found to be related to dough physical properties (27), and here, we employed FTIR spectroscopy to examine the protein secondary structure in dough made with different grades of flours (Fig. 2C–E) and those made with wPDI addition (Fig. 2C–E, dashed lines). Assignment of the amide I band in the above second-derivative spectra of each dough sample indicated that the α-helix and β-turn contents decreased, while the β-strand content increased after wPDI addition (Fig. 2F).

### wPDI recruits HMW-GS 1Dx5 for gluten network formation

**Fig. 3.**
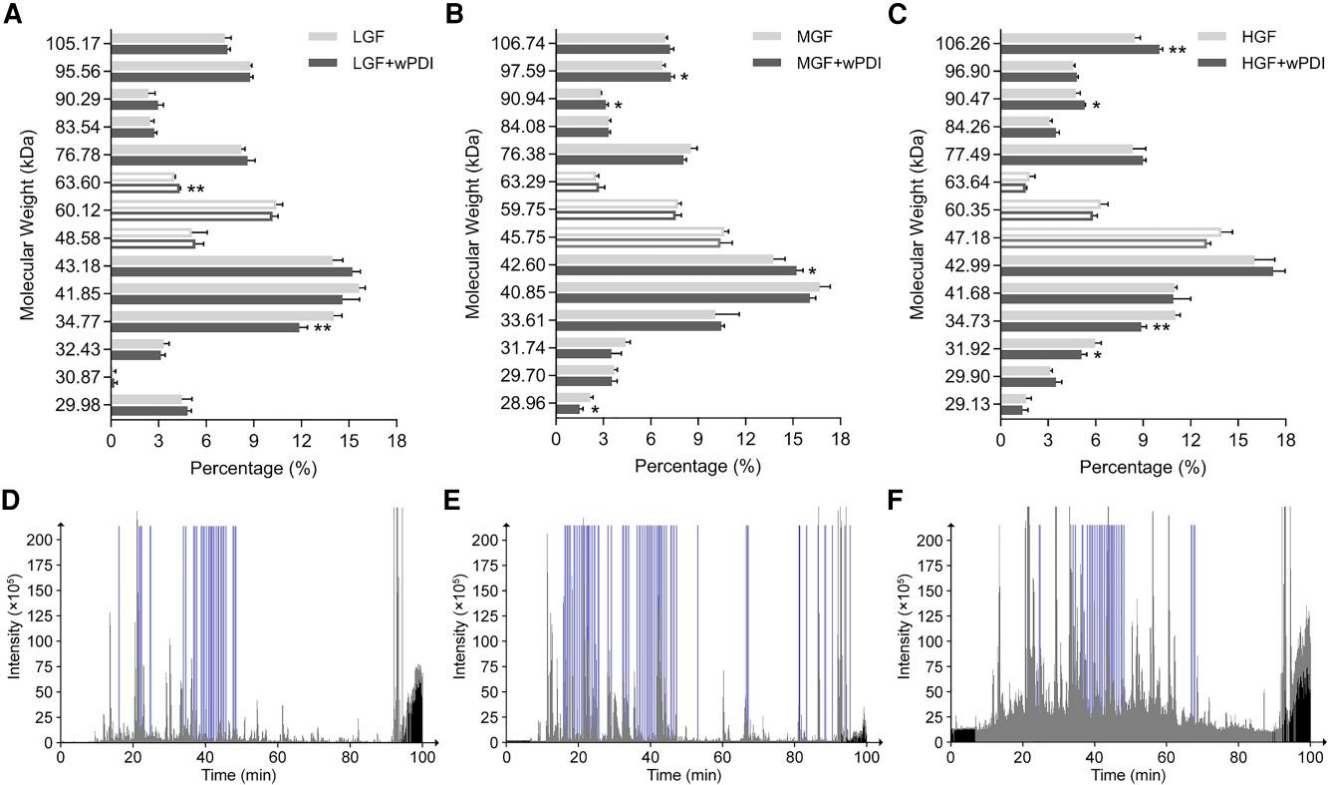
Participation and identification of proteins in the formation of the gluten network induced by wPDI. A–C) Molecular weight calculation and protein band quantification of proteins involved in the gluten network formation, without and with the addition of wPDI, of dough made with LGF, MGF, and HGF, respectively. The SDS-insoluble proteins, which generally participate in gluten network formation, are analyzed with reducing SDS‒PAGE. Each band density is quantified, and the corresponding molecular weight is identified with standard protein markers. Filled bars represent HMW and LMW glutenins, respectively, and empty bars represent gliadins. *** indicates *P* ≤ 0.001, ** indicates 0.001 < *P* ≤ 0.01, and * indicates 0.01 < *P* ≤ 0.05. D) LC‒MS/MS identification of the 90.47-kDa glutenin bands significantly increased their participation in the gluten network formation induced by wPDI in the dough made with HGF. Gray is the raw HPLC chromatogram of trypsin-digested protein fragments, and blue represents the predicted chromatogram of HMW-GS 1Dx5. E) LC‒MS/MS identification of the 106.26-kDa glutenin bands in dough made with HGF. F) LC‒MS/MS identification of the 97.59-kDa glutenin bands in dough made with MGF. Blue indicates the predicted chromatogram of HMW-GS 1Dx5, and gray is the experimentally obtained HPLC chromatogram.

To determine which gluten protein(s) were affected most by wPDI during gluten network formation, the GMP of each dough sample was quantitatively analyzed with reducing SDS‒PAGE and gel images were subjected to automatic band recognition, molecular weight calculation, and density quantification (Fig. S2A–C). The following groups of proteins were found in GMPs: those with molecular weights higher than 67 kDa (HMW-GSs), those with molecular weights lower than 45 kDa (LMW-GSs), and those in between (ω-gliadins) (11). The band density of each protein was compared with its corresponding band from dough sample with wPDI addition and quantitatively analyzed. Based on the results, the participation of 63.6 kDa gliadin in GMP was significantly increased in dough produced with LGF (Fig. 3A). For LMW-GSs, the participation of 34.77 kDa in dough made of LGF (Fig. 3A), 28.96 kDa in dough made of MGF (Fig. 3B), and 31.92 and 34.73 kDa in dough made of HGF (Fig. 3C) significantly decreased in GMP after wPDI was added. On the other hand, LMW-GSs of 42.60 kDa significantly increased in GMP formation after wPDI addition (Fig. 3B). For HMW-GSs, the ∼90 kDa proteins in dough made of MGF and HGF (Fig. 3B and C) showed significantly increased participation in GMP. Additionally, HMW-GS of 97.59 kDa in dough made of MGF (Fig. 3B) and of 106.26 kDa in dough made of HGF (Fig. 3C) showed significantly increased participation in GMP after wPDI addition.

As we were concerned about how wPDI strengthened gluten network formation, protein bands of decreased participation were less relevant than these with increased participation. It is reported that HMW-GSs were playing more important role in the gluten network formation (24). Thus, the 90.47-kDa band in dough made of HGF, the 97.59-kDa band in dough made with MGF, and the 106.26-kDa band in dough made with HGF were digested and subjected to LC‒MS/MS analysis. Chromatograms of the digested protein fragments were compared to those of the in silico digested fragments of the complete *Triticum aestivum* proteins in the UniProt database (UniProt_wheat_2022). For the HMW-GS band of ∼90 kDa, 470 proteins were identified (Table S1), and the predicted protein with a similar molecular weight was HMW-GS 1Dx5 which has a molecular weight of 90.2 kDa. For the HMW-GS band of 97.59 kDa, 243 proteins were identified (Table S2). For the HMW-GS band of 106.26 kDa, 288 proteins were identified (Table S3). However, no HMW-GS of similar molecular weight was found among them. We then compared the chromatogram of the digested protein and the predicted chromatogram of in silico digested HMW-GS 1Dx5 via thermomsf-parser (28). The predicted chromatogram of HMW-GS 1Dx5 overlaid well with the experimental chromatogram of the 90-kDa protein (Fig. 3D), while another predicted HMW-GS 12 did not (Fig. S3A). We also compared the predicted chromatogram of all HMW-GSs found, regardless of their molecular weight, with those of 106.26 and 97.59 kDa proteins. Interestingly, HMW-GS 1Dx5 overlaid well with these proteins (Fig. 3E and F). For the 106.26-kDa protein, the overlay of HMW-GS PW212 and D10 was poorer than that of HMW-GS 1Dx5 (Fig. S3B and C). For the 97.59-kDa protein, HMW-GS DY10 did not overlay better than 1Dx5 (Fig. S3D). Although it is not sure how HMW-GS 1Dx5 was identified in protein bands with higher molecular weights, these results suggested that HMW-GS 1Dx5 was most likely more recruited into gluten network formation in the presence of wPDI.

### wPDI interacts directly with HMW-GS 1Dx5

**Fig. 4.**
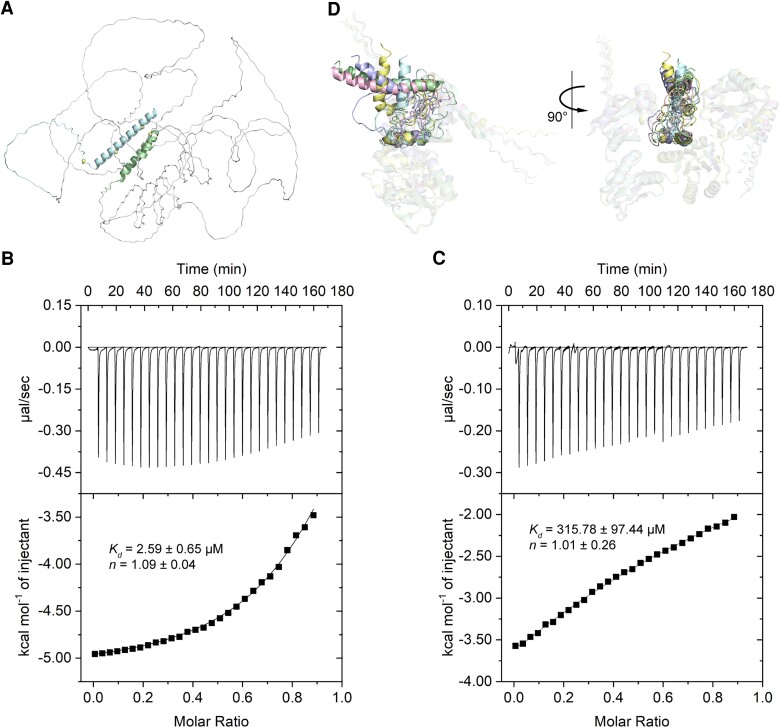
HMW-GS glutenin 1Dx5 and its interaction with wPDI. A) The 3D structure model of full-length glutenin 1Dx5 predicted by AlphaFold 3. Pale cyan indicates the N-terminal domain, pale green indicates the C-terminal domain, pale yellow spheres indicate the cystine residues, and gray indicates the central repetitive domain. B,C) Thermograph of isothermal titration of HMW-GS 1Dx5-NTD into reduced and oxidized wPDI, respectively. D) Interaction between HMW-GS 1Dx5-NTD and wPDI predicted by AlphaFold-Multimer. The top five poses of HMW-GS 1Dx5-NTD interacting with wPDI (transparent) are shown in different colors, including pale green, light blue, pale yellow, light pink, and pale cyan.

To determine how wPDI affected the participation of HMW-GS 1Dx5 in gluten network formation, we initially planned to study their molecular interactions. HMW-GS 1Dx5 contains an 89-amino-acid-residue N-terminal domain (1Dx5-NTD) and a 42-amino-acid-residue C-terminal domain flanking a 681-amino-acid-residue central repetitive domain (Fig. S4A). Unfortunately, the whole-length HMW-GS 1Dx5 was expressed in the form of inclusion bodies and disenabled us to study its molecular interaction with wPDI. The 3D structure of HMW-GS 1Dx5 was predicted with AlphaFold 3, and there was little defined secondary structure of the central repetitive sequence (29). Both the N-terminal and C-terminal domains of HMW-GS 1Dx5 were predicted mainly α-helical with three and one cysteine residues in the N- and C-terminal domain, respectively (Fig. 4A). Since intermolecular crosslinking between these cysteine residues contributes to the gluten matrix formation and due to these multiple cystines in 1Dx5-NTD, this domain is subcloned and purified for the subsequent study. Additionally, due to the predicted unstructured feature of the central repetitive domain, it is not considered as a priority to subcloning and purification.

PDI, on the other hand, interacts with substrates that exhibit different redox states and corresponding configurations (30). Here, wPDI of both oxidized and reduced states was subjected to isothermal titration calorimetry analysis with 1Dx5-NTD. 1Dx5-NTD^AAA^, a triple mutant in which all three cysteines were mutated into alanine, was generated with site-directed mutagenesis to discern the heat involved during their binding from that of 1Dx5-NTD self-crosslinking (Table S4). Titration of 1Dx5-NTD^AAA^ into reduced wPDI (wPDI^R^) yielded a dissociation constant of 2.59 ± 0.65 μM (Fig. 4B), and the thermodynamic measurements suggested an entropy-driven, more hydrophobic interaction between them (Fig. S5A). In contrast, titration of 1Dx5-NTD^AAA^ into oxidized wPDI (wPDI^O^) yielded a dissociation constant of 315.78 ± 97.44 μM (Fig. 4C) and an enthalpy driven by hydrogen bonding and van der Waals interactions between them (Fig. S5A).

### wPDI catalyzes HMW-GS 1Dx5 crosslinking by altering its folding

**Fig. 5.**
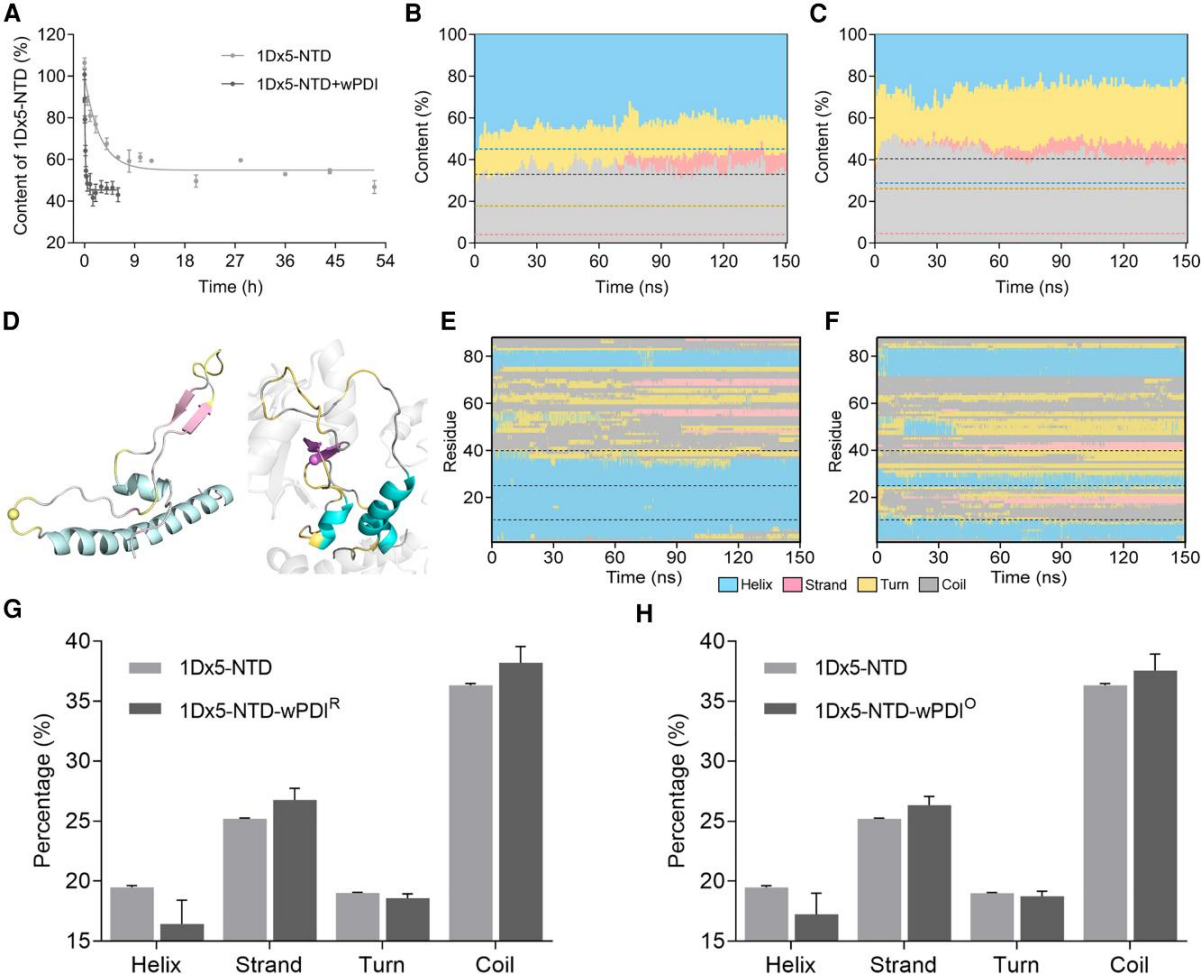
Glutenin 1Dx5-NTD crosslinking and its structural change chaperoned by wPDI. A) Crosslinking of HMW-GS 1Dx5-NTD measured by quantifying the decrease of protomers by nonreducing SDS‒PAGE. Lines represent a one-phase exponential fit of the data. B,C) Secondary structure dynamics in HMW-GS 1Dx5-NTD without and with the chaperoning of wPDI, respectively. The *y*-axis represents secondary structure contents, and the *x*-axis represents simulation time. Blue indicates α-helix, pink indicates β-strand, yellow indicates β-turn, and gray indicates coil. Dashed lines represent the average portion of each secondary structure element, blue indicates α-helix, brown indicates the sum of β-strand and turns, and gray indicates coil. D) A snapshot of 1Dx5-NTD without (Left) and with (Right) the chaperoning of wPDI. In the absence of wPDI, α-helix, β-strand, β-turn, and coil are shown in pale cyan, light pink, pale yellow, and gray70, respectively. In the presence of wPDI, these structures are shown in cyan, light magenta, yellow‒orange, and gray90, respectively. Sphere indicates the *C*_α_ of C40. E,F) Secondary structure distribution in 1Dx5-NTD without and with the chaperoning of wPDI, respectively. The *y*-axis represents amino acid residues, and the *x*-axis represents simulation time. Different colors indicate the same as above. G) Calculated secondary structure contents of 1Dx5-NTD in the absence and presence of wPDI^R^ from its CD spectrum. H) Calculated secondary structure contents of 1Dx5-NTD in the absence and presence of wPDI^O^ from its CD spectrum.

To investigate how wPDI-promoted HMW-GS 1Dx5 crosslinking, the self-crosslinking reaction rate of wild-type 1Dx5-NTD, on which three cystine residues remained, was determined. The reaction was performed in either the absence or the presence of wPDI and terminated at different time intervals, and the remaining 1Dx5-NTD protomers were quantified by SDS–PAGE (Fig. S5B and C). Residual protomers were fitted to one-phase decay, and the reaction rate for 1Dx5-NTD crosslinking in the absence of wPDI was 1.31 × 10^3^ s^−1^, while that in the presence of wPDI increased to 2.42 × 10^4^ s^−1^ (Fig. 5A).

To investigate the mechanism behind the catalyzed crosslinking of 1Dx5-NTD, we further simulated the molecular dynamics between its interaction with wPDI. Despite much effort, the crystal structure of wPDI was not obtained, and we used AlphaFold to predict the wPDI structure instead. The predicted wPDI model contains four typical theoredoxin domains (Figs. S4B and S6A), similar to that of human PDI (hPDI). When we compared the wPDI model with the oxidized hPDI (hPDI^O^) and reduced hPDI (hPDI^R^) structures, the wPDI model yielded a conformation closer to the hPDI^O^ structure with a residual mean square deviation (RMSD) of 4.523 Å (Fig. S6B). On the other hand, there was a larger *en bloc* movement of the a′ domain between the wPDI model and the hPDI^R^ structure, with an RMSD of 7.579 Å (Fig. S6C).

The interaction between 1Dx5-NTD and wPDI was predicted with AlphaFold-Multimer, and different binding patterns were observed between 1Dx5-NTD and wPDI (Fig. 4D). Among them, the molecule with the least interaction between the well-folded α-helix and wPDI was subjected to molecular dynamics simulation. Our 150 ns simulation reached a structural equilibrium in which the structure of 1Dx5-NTD in the presence of wPDI fluctuated much less than that in the absence of wPDI, as suggested by its lower RMSD (Fig. S7A). Additionally, the 1Dx5-NTD molecule yielded a slightly more compacted structure in the presence of wPDI (Fig. S7B). When the secondary structure contents were examined, it was found that part of 1Dx5-NTD could fold into β-strands (Fig. 5B and Fig. S7C and D). In contrast, the frequency of β-strands was much higher when wPDI was present (Fig. 5C). Over the simulation, the average content of β-strands increased from 4.12 to 4.66% and the α-helix structure decreased from 45.12 to 28.f75% when wPDI was present (Fig. 5C). Significant structural alteration occurred when 1Dx5-NTD was bound to wPDI, and a structural deviation of 17.292 Å was observed (Fig. 5D). Structural change at residue resolution was examined, and C40 exhibited increased occurrence in the β-strand from a coil structure (Fig. 5D–F). This result was verified with circular dichroism (CD); in the presence of either wPDI^R^ (Fig. 5G) or wPDI^O^ (Fig. 5H), the β-strand content increased in 1Dx5-NTD compared to that obtained without the presence of wPDI (Fig. S7E and F).

## Discussion

Although most evidence supports the positive role of wPDI in promoting gluten network formation (12–16), a slightly different perspective also exists (17). To clarify, we systematically employed almost all golden-standard physical measurements to determine how “in vitro” gluten network formation was affected by wPDI with dough made of various flours. To identify all possible substrate proteins of wPDI, a full spectrum of flours with all grades and thus grounded from different wheat cultivars were used. Our results were consistent with expectations that higher protein content correlated with stronger dough formation (31). More importantly, addition of wPDI further improved its farinographic, rheologic, and alveographic characteristics in the same dough. For the first time, 3D reconstructed gluten networks with and without wPDI addition were generated and analyzed. Quantitative analysis indicated that wPDI addition further strengthened the formation of gluten networks at a fixed protein content. Additionally, an increase in the protein percentage area and “total protein length” suggested that wPDI promoted more proteins participated in the gluten network as verified by the increase of GMP portions. On the other hand, an increase in junction density indicates that more proteins participated in organized protein knitting, suggested that wPDI promoted an ordered protein organization into a gluten network. This result was consistent with the decrease of free sulfhydryl groups in dough where wPDI very likely exhibited oxidase activity. This evidence supported a robustly established and unambiguously defined role of wPDI in promoting gluten network formation.

Gliadins are often monomeric gluten proteins with intramolecular disulfide bonds and are considered to contribute to dough viscosity (16). Here, we show that increased viscosity is accompanied by a more organized gluten network, indicating that gliadins are simultaneously involved in gluten network formation with glutenin. Since gliadins are not crosslinked into the gluten network, their increased participation is more likely to be noncovalent just like starch granules. Allelic variations in *Glu-1* lead to many different glutenins expressed in the endosperm of wheat, and we identified that HMW-GS 1Dx5 is most likely (but not exclusively) the substrate of wPDI at least “in vitro.” The addition of wPDI slightly, but statistically significantly, increased HMW-GS 1Dx5 participation in the gluten network formation which eventually led to measurable physical changes in the dough. This result was consistent with previous findings that HMW-GS 1Dx5 and 1Dy10 contribute mostly to the physical properties of dough (32, 33), and it was later demonstrated that HMW-GS 1Dx5 contributed more than 1Dy10 (34, 35). Although difficulties in purification of the whole-length HMW-GS 1Dx5 blinded us from seeing its interaction with wPDI, the crosslinking reaction certainly happens within its NTD where cysteine residues located. Purified 1Dx5-NTD exhibited ∼18.5-fold accelerated crosslinking rate in the presence of wPDI, which was consistent with the moderate catalysis capacity of PDI in oxidative folding in vitro (4). This mild oxidase activity correlated with the slightly improved gluten network characters. Compared with wPDI^O^, 1Dx5-NTD exhibited an ∼122-fold increased affinity with wPDI^R^ via majorly hydrophobic interaction. On the contrary, it exhibited majorly polar interactions with wPDI^O^ with decreased binding affinity. These results indicated that 1Dx5-NTD was possibly folded by wPDI^R^ first to reach an appropriate conformation before being oxidized by wPDI^O^.

The correlation between dough total secondary structures and physical properties revealed that higher β-strand contents are associated with better physical properties of dough (27, 36–38). Consistently, our FTIR results indicated that wPDI promoted β-strand formation in the dough, and our CD results indicated that wPDI folded 1Dx5-NTD into its conformation with a higher β-strand content. Our simulation results suggested that wPDI specifically folded C40 into β-strand structure. The mechanism by which β-strand structure promotes disulfide crosslinking is unclear, but in a recent survey on intermolecular disulfide bonds in protein crystal structures deposited in the Protein Data Bank, it was found that disulfide bonds tend to form between cysteines in β-strands, and intermolecular bonds are more likely to form between proteins with the same structure class (39). Nonetheless, the chaperone activity of wPDI is involved in the promoted folding of 1Dx5-NTD. Of course, the overall effect of wPDI working on the whole-length HMW-GS 1Dx5 needs further investigation.

Here, from every angle, it is demonstrated that wPDI could promote the formation of gluten networks, thus improving the physical properties of dough. The major (but not exclusive) substrate of wPDI is HMW-GS 1Dx5, and wPDI folds its N-terminal domain conformation into β-strand structures that favors crosslinking between cysteines. As glutenins in the flour of grounded wheat are chaperoned and oxidized by wPDI, wPDI is a significant molecular breeding target for wheat of better quality and a promising biological dough improver.

## Materials and methods

### Protein expression and purification

Plasmids encoding wPDI (pET-30b-*wpdi*) or 1Dx5-NTD (pET-30b-*1dx5-ntd*) were transformed into *E. coli* BL21 (DE3) and cultured in LB medium with 50 μg/mL kanamycin at 37°C in a shaking incubator. When the OD_600_ reached 0.6–0.8, wPDI expression was induced by adding 0.5 mM isopropyl β-D-thiogalactoside (IPTG) and 1Dx5-NTD was induced by adding 1 mM IPTG at 20°C overnight. Cells were harvested by centrifugation at 4,000 rpm for 30 min before lysis by sonication. Cell debris was removed by centrifugation at 12,000 rpm for 30 min, and the supernatant was filtered through a 0.22-μm syringe filter before being loaded onto a Ni-NTA affinity column preequilibrated with a buffer containing 50 mM Tris (pH 8.0) and 500 mM NaCl. Proteins were eluted with the same buffer that contained increasing concentrations of imidazole. Elution fractions were examined with SDS‒PAGE, and 1Dx5-NTD usually reached a single band, while further purification was needed for wPDI. On a HiTrap^TM^ Q FF (GE Healthcare) anion exchange column preequilibrated with 20 mM PBS (pH 6.5), 50 mM NaCl, and 2.5 mM ethylene diamine tetraacetic acid (EDTA). wPDI was loaded and then eluted with the same buffer containing increasing concentrations of NaCl. The fractions were examined with SDS–PAGE and pure wPDI was collected and concentrated. All the proteins were dialyzed into a 20-mM PBS buffer (pH 7.4) unless otherwise mentioned.

### Dough preparation and its farinographic, rheologic, and alveographic characterization

High-, medium-, and low-gluten wheat flours were purchased from Beijing Guchuan Foods Co. Ltd. (Beijing, China), and the protein content of each was determined according to the ISO 20483:2013 by the Kjeldahl method (40). Dough was formed by mixing 250 g flour with a solution containing the indicated concentration of wPDI dialyzed against 5 mM PBS buffer (pH 7.5).

The farinographic properties of each dough were measured with Mixolab (Chopin Technologies, Paris, France). In total, 75 g of dough was mixed in a bowl with an early torque of 1.1 ± 0.05 N·m at 80 rpm, and the real-time torque values were then recorded as the dough was mixed with the following programmed temperature cycle: hold at 30°C for 30 min, increase to 90°C at 4°C/min and hold for 7 min, and decrease to 50°C at 4°C/min and hold for 5 min. The maximum torque at the initial mixing stage was recorded as C1, while the development time was the time needed for the torque to reach C1. The stability time was the time needed for the torque to remain in the 89–111% range of C1.

The rheologic properties of each dough were measured with a Discovery Hybrid Rheometer (TA Instrument, New Castle, DE, USA). Dough was placed on the testing plate with a gap size of 1,000 μm between the flat plate (40 mm in diameter) and the test plate. Excessive dough outside the plate edge was removed after loading, and paraffin oil was applied to cover the lateral surface to prevent dryness. An amplitude scan was carried out at strain values of 0.01–1% at a constant frequency (1 Hz, 25°C) within the linear viscoelastic region. The oscillation test was carried out at a target stress of 0.1% and scanning frequency of 0.1–100 Hz at 25°C.

The alveographic properties of each dough were measured with an Alveolab 1/2 instrument (Chopin Technologies, Paris, France) principally the same as previously described (16). The flour–enzyme solution mixture was mixed for 8 min at 24°C, and the resulting dough was forced through the extrusion gate as a strip of 0.4 cm. The strip of a certain length was rolled with a pin to a uniform thickness of ∼0.4 cm, cut into a circular disk, transferred to an oiled steel plate, and subjected to a rest period of 28 min at 25°C in a tempered compartment. Afterward, each circular dough piece was placed on metal plates for automatic alveographic measurements in a chamber with a humidity of 65% at 20°C. Measurements were the peak height (P in mmH_2_O) of the dough representing dough tenacity and the work necessary to deform the dough bubble (W in joules). Five replicates of each dough sample were repeated.

### Gluten network imaging and analysis

Dough samples were prepared with an addition of rhodamine B at a final concentration of 0.01 ppm (Macklin, Shanghai, China). Approximately 2.0 g of dough was transferred to an object carrier, sealed with a cover glass, and observed with an Olympus IX83 inverted microscope equipped with a FluoView FV1200 confocal system (Olympus, Tokyo, Japan) and a 60× objective lens (UPlanSApo 60×, numerical aperture = 1.35, working distance = 0.17). The excitation wavelength was set to 559 nm, and emission at 603 nm was detected. For each sample, different areas on the *x–y*-axis were recorded and dough samples were produced in triplicate. For 3D visualization, acquisition was performed in 0.5 μM *z*-steps and the Imaris software package (Bitplane AG, Zurich, Switzerland) was used to visualize z-stack and reconstruct the 3D architecture of the samples. The gluten network images were analyzed with AngioTool64 v. 0.6a (National Cancer Institute, NIH, MD, USA) to obtain the parameters of protein percentage area, total protein length, and junction density as described previously (3).

### Glutenin distribution analysis

Dough samples were frozen at −80°C for 12 hours, freeze-dried in a lyophilizer, and then ground into powders. Albumins, globulins, and partial gliadins were removed by dissolving 100 mg samples in 2% (w/v) NaCl solution, vortex mixing every 5 min for 30 min, and centrifuging at 10,000*×g* for 5 min. The clarified supernatant was discarded, and the residual pellet was resuspended in 70% (v/v) ethanol and centrifuged again, and the supernatant was discarded to partially remove gliadins. SDS-soluble proteins were extracted by resuspending the residual pellet in 0.5% (w/v) SDS buffer containing 50 mM PBS (pH 6.9) for 30 min with vortex mixing every 5 min. After centrifugation, the supernatant contained SDS-soluble proteins. The residual pellet was resuspended in the same SDS buffer, sonicated at 100 W for 60 s, and vortexed and clarified by centrifugation, and the supernatant contained SDS-insoluble proteins. The SDS-soluble and SDS-insoluble protein concentrations were determined with a BCA protein assay kit (Biosharp Life Sciences, Hefei, Anhui, China) (26).

### Sulfhydryl content analysis

Dough was prepared as described above and washed repeatedly with water to remove starch until the water tested negative with iodine. The remaining gluten protein was frozen at −80°C for 12 h, freeze-dried, and ground into fine powders. 15 mg of this powder was dissolved in 1.5 mL buffer A containing 0.2 M Tris–HCl (pH 8.0), 3 mM EDTA, 1% SDS, and 8 M urea and incubated at room temperature for 1 h. Buffer B of 0.15 mL [buffer A containing 10 mM 5,5′-dithiobis(2-nitrobenzoic acid) (DNTB)] was added to each above solution, mixed, incubated for 25 min, and centrifuged at 13,600*×g* for 10 min, and its absorbance at 412 nm was measured on a DeNovix DS-11 FX+ spectrophotometer (DeNovix Inc., Wilmington, DE, USA) at 412 nm with a 1-cm cuvette. The content of free sulfhydryl groups was calculated as described previously (16).

### Gluten protein total secondary structure analysis

Gluten protein was prepared as described above, lyophilized, ground into a fine powder, mixed with 100-fold dry potassium bromide, pressed into transparent round tablets, and tested on a PE400 FTIR spectrometer (PerkinElmer, Waltham, MA, USA). A total of 64 scans were collected at an interval of 4 cm^−1^ from 4,000 to 400 cm^−1^, and the spectra were deconvoluted by a nonlinear regression curve fitting program of Gaussian peaks with PeakFit v4.12 (SPSS, Chicago IL, USA). The amide I region (1,600 – 1,700 cm^−1^) was subjected to secondary structure content analysis as described previously (27).

### Glutenin analysis and identification

The SDS-insoluble proteins in the above experiment section were analyzed with reducing SDS–PAGE. The samples were electrophoresed at a voltage of 80 V for the 5% stacking gel and 120 V for the 12% resolving gel until the bromophenol blue dye reached the bottom of the gel and stained with Coomassie brilliant blue to visualize the proteins. The protein bands were captured with a SageCreation ChampGel 5000 gel documentation system (Beijing Sage Creation Science Co. Ltd, Beijing, China). The molecular weight and each band density were calculated with its related SageCapture software calibrated with TrueColour Prestained Protein Markers (Sangon Biotech, Shanghai, China). The experiment was repeated in triplicate, and the protein bands with the most significant intensity increase were subjected to protein identification as described below.

The protein band was destained, reduced with dithiothreitol (DTT), alkylated with iodoacetamide, digested with trypsin (Promega, USA) overnight, acidified with trifluoroacetic acid (TFA), dissolved in acetonitrile (ACN), separated with a reversed-phase column (75 μm × 20 cm, 3 μm) packed in-house (ReProsil-Pur C18 AQ, Dr Maisch GmbH, Germany), eluted with two solvent buffers (buffer A: 99.9% H_2_O and 0.1% TFA and buffer B: 99.9% ACN and 0.1% TFA), and analyzed with a nanoLC-Q Exactive Orbitrap Mass Spectrometer (Thermo Fisher Scientific, USA). The full scan was processed from 300 to 1,600 m/z, and dynamic exclusion was employed for 40 s. Proteins were identified against the complete wheat proteins in the UniProt database (UniProt_wheat_2022) using Proteome Discoverer (1.4.0.288) with the following settings: a maximum of two missed cleavages were allowed, carbamido methylation of cysteines was considered a fixed modification, and oxidation of methionine and protein N-terminal acetylation were classified as variable modifications. The false discovery rate (FDR) was <1%.

### Isothermal titration calorimetry

On a MicroCal VP-ITC calorimeter (Microcal Inc., Northampton, MA, USA), 0.3 mM 1Dx5-NTD^AAA^ triple mutant protein was titrated into the same buffer containing 70 μM wPDI with 27 successive additions of 10 μL of 1Dx5-NTD^AAA^ solution and an interval time of 360 s. The first titration was performed with 2 μL titrant. Titration of wPDI^R^ was performed in the presence of 1 mM DTT in both buffers, and titration of wPDI^O^ was performed in the presence of 0.5 mM oxidized glutathione (GSSG) in both buffers. Thermographs were integrated with ORIGIN software (Microcal Inc.) by plotting the heat change against the addition of 1Dx5-NTD^AAA^ into wPDI. Data were best fitted by a single-site binding model, and stoichiometry and affinity values were calculated accordingly.

### Glutenin crosslinking assay

Both 1Dx5-NTD and wPDI were dialyzed against a buffer containing 100 mM Tris–HCl (pH 8.0), 50 mM NaCl, and 2 mM EDTA. 1Dx5-NTD was reduced with 10 mM DTT, and the residual DTT was removed by a PD-10 column (GE Healthcare). Freshly reduced 1Dx5-NTD at 50 μM was oxidized by 0.2 mM GSSG, and an aliquot of the reaction was stopped by adding iodoacetamide at a final concentration of 2 mM into the solution. 0.67 μM wPDI was added into another set of parallel solutions, and the same procedure was performed. Finally, the crosslinking products were examined with nonreducing SDS–PAGE, stained with Coomassie brilliant blue, and captured and quantified as described above. The band density of residual 1Dx5-NTD protomers was fitted with the one-phase exponential decay model with GraphPad Prism (version 8.0, GraphPad Software Inc., La Jolla, CA, USA).

### Circular dichroism

5 μM 1Dx5-NTD protein with and without the presence of 1 μM wPDI was placed into a quartz cuvette with a 1-mm path length. CD spectra of each sample were recorded on a Chirascan CD polarimeter (Applied Photophysics, UK) by scanning the sample from 200 to 260 nm at 25°C with a bandwidth of 1 nm and step size of 1 nm at a scanning rate of 30 nm/min. The spectrum of 1Dx5-NTD was obtained by subtracting the spectrum of the solution containing the same concentration of wPDI from the spectrum of the 1Dx5-NTD/wPDI mixture solution.

### Protein structure prediction and interaction simulation

The wPDI structure and its interaction with 1Dx5-NTD were modeled using the ColabFold platform (41). The molecular dynamics of 1Dx5-NTD alone and its interaction with wPDI were simulated. The CHARMM36 force field was employed to describe all standard residues in the wPDI-1Dx5-NTD complex (42). Sodium ions were used to neutralize the system, which was explicitly solvated using the TIP3P water potential in a box with a minimum clearance of 10 Å between the solute and the side of the box (43). Particle Mesh Ewald molecular dynamics CUDA version was employed to run the molecular dynamics simulation on Graphic Processing Unit (GPUs) with the GROMACS 2020.3 package (44, 45). Energy minimization was initiated for 5,000 steps to repair the asymmetrical geometries in the system with a determined force of 1,000.0 kJ/mol/nm. The Particle Mesh Ewald method was performed for electrostatic corrections, whereas the geometry of the bond length was confined using the steepest descent algorithm method. The NVT (temperature) and NPT (pressure) systems were fixed at 300 K and 1.0 bar after energy minimization, and each was performed for 50,000 ps. Temperature and pressure normalization was conducted with the Parrinello–Rahman barostat method, water molecule geometry was restrained with the SETTLE algorithm, and the nonwater bonds were restrained using the LINCS algorithm. Finally, an unrestrained 150-ns run was carried out under the same conditions in the equilibrium step, and the trajectory snapshots were saved every 10 ps. Built-in modules in GROMACS were used to analyze the data (46, 47).

## Supplementary Material

pgae356_Supplementary_Data

## Data Availability

All data are available within this article and its supplementary information.

## Supplementary Material

Figures S1–S7 and Tables S1–S4 are available.
