# Supplementary material for: Identification and mechanism of wheat protein disulfide isomerase-promoted gluten network formation
Source: PNAS Nexus. 2024 Aug 21;3(9):pgae356. doi: 10.1093/pnasnexus/pgae356 (PMC11376372; doi:10.1093/pnasnexus/pgae356)
Supplement: pgae356_Supplementary_Data [file pgae356_supplementary_data.zip › PNASNEXUS-PNASNEXUS-2024-00980-s05.docx]

**Supplementary Table S4**

Table S4 Primers used to construct 1Dx5-NTD mutants

| Primer Name | Primer Sequences （5'-3'） |
| --- | --- |
| C10A-Forward | TCTGAGCAACTACAGGCTGAGCGCGAG |
| C10A-Reverse | GCCTGTAGTTGCTCAGAGGCCTCACCT |
| C25A-Forward | CGCGAGCTCAAGGCAGCCCAGCAGGTC |
| C25A-Reverse | GCTGCCTTGAGCTCGCGCTCCTGGAGC |
| C40A-Forward | GACATTAGCCCCGAGGCCCACCCCGTC |
| C40A-Reverse | GCCTCGGGGCTAATGTCTCGGAGCTGT |
