# Supplementary material for: Identification and mechanism of wheat protein disulfide isomerase-promoted gluten network formation
Source: PNAS Nexus. 2024 Aug 21;3(9):pgae356. doi: 10.1093/pnasnexus/pgae356 (PMC11376372; doi:10.1093/pnasnexus/pgae356)
Supplement: pgae356_Supplementary_Data [file pgae356_supplementary_data.zip › PNASNEXUS-PNASNEXUS-2024-00980-s06.docx]

**Supporting Information**

Supporting information contains:

Supplementary Fig. S1-S7

Supplementary Table S1-S4

**Supplementary Figures and Legends**

**
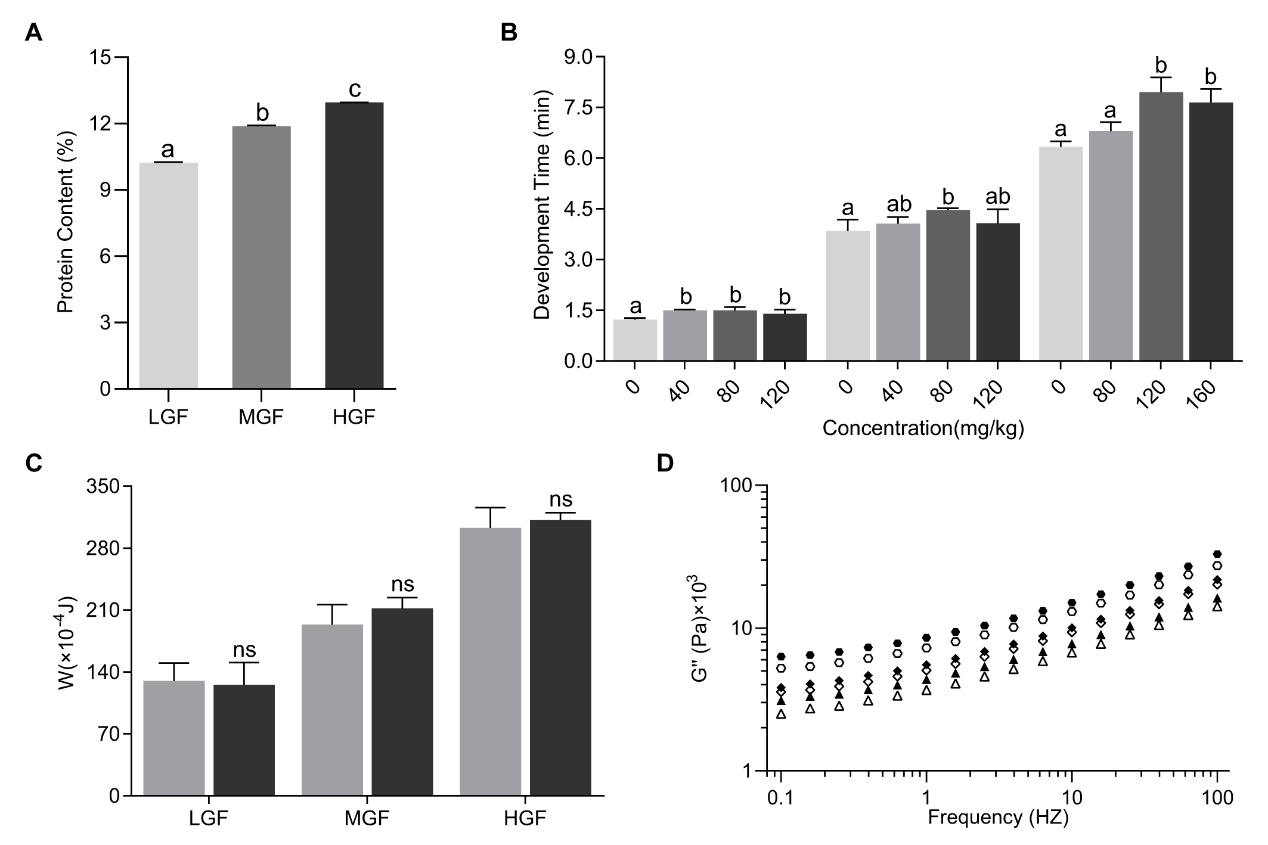
**

**Supplementary Fig. 1| Additional macro properties of dough with wPDI addition. A,** Measured protein contents of LGF, MGF, and HGF used in this study and different letters represent significant differences (*p* < 0.05). **B,** Development time, another representative farinographic character, of dough made with LGF (left group), MGF (middle group), and HGF (right group). Dough with 0, 40, 80, and 120 mg/kg wPDI addition is compared. **C,** Dough baking force, another representative alveographic character, in the absence and presence of wPDI added to dough made with LGF, MGF, and HGF. Dough with added wPDI is shown in grey and dough without wPDI is shown in black. ns stands for not significant. **D,** Viscous moduli, another representative rheologic character, of dough made with LGF (triangles), MGF (diamonds), and HGF (hexagon). Dough added with wPDI is shown in filled symbols, and without wPDI is shown in empty symbols.


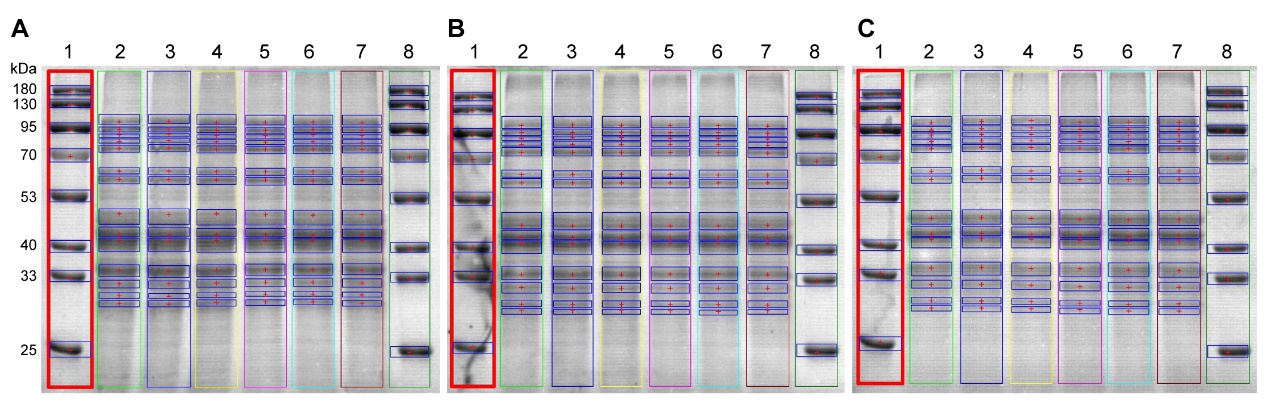


**Supplementary Fig. 2| SDS‒PAGE analysis on the participation of glutenin in the formation of gluten networks induced by wPDI.** The GMP protein is subjected to reducing SDS‒PAGE analysis. **A**-**C,** Reducing SDS‒PAGE of GMP proteins of dough made with LGF, MGF, and HGF, respectively. Lanes 1 and 8 are standard protein markers, lanes 2-4 are replicates of SDS-insoluble proteins extracted from dough without wPDI addition, and lanes 5-7 are replicates of SDS-insoluble proteins extracted from dough with wPDI addition. Squares with red crosses indicate protein bands automatically recognized and quantified by SageCapture^TM^ software.


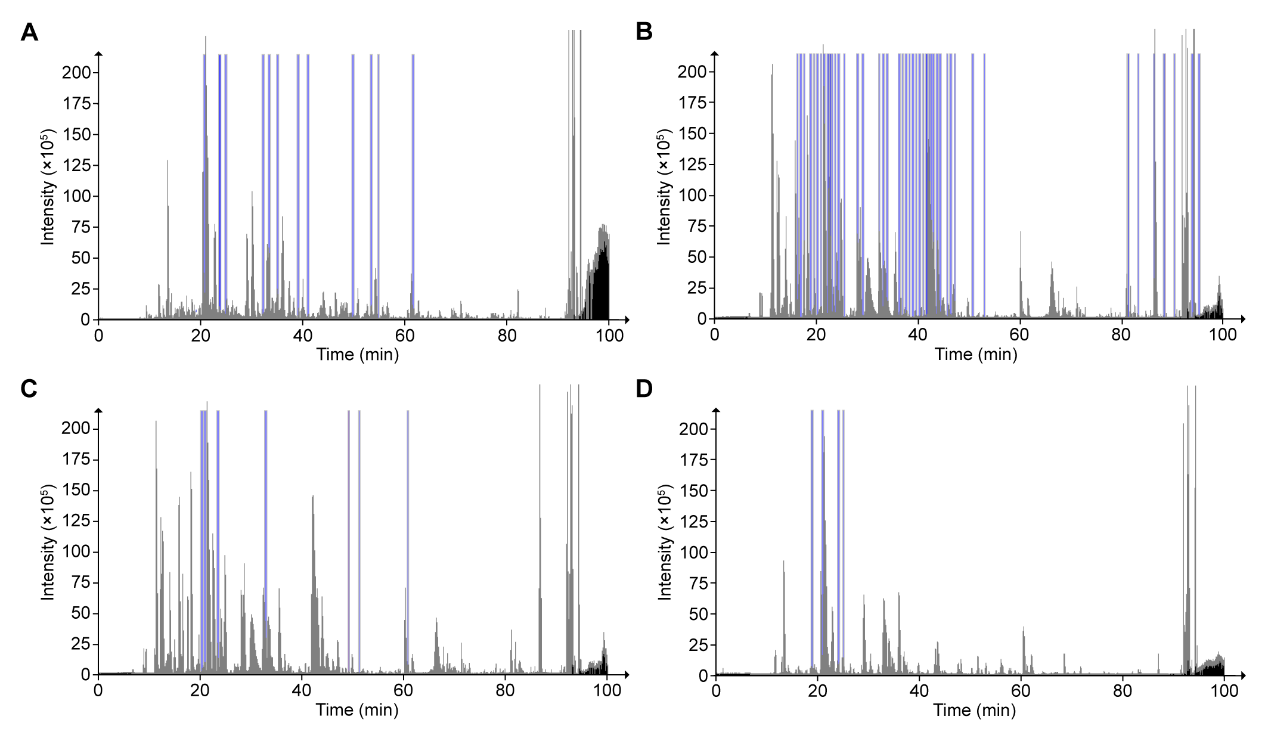


**Supplementary Fig. 3| LC‒MS/MS analysis of glutenins significantly involved in the gluten network formation induced by wPDI.** LC‒MS/MS identification of proteins significantly increased their participation in the gluten network formation induced by wPDI. Grey is the raw HPLC chromatogram of trypsin-digested protein fragments, and **A,** blue indicates the predicted chromatogram of HMW-GS 12 in the protein band of 90.47 kDa of the gluten network made with HGF. **B,** Blue indicates the predicted chromatogram of HMW-GS PW212 in the protein band of 106.26 kDa of the gluten network made with HGF. **C,** Blue indicates the predicted chromatogram of HMW-GS DY10 in the protein band of 106.26 kDa of the gluten network made with HGF. **D,** Blue indicates the predicted chromatogram of HMW-GS DY10 in the protein band of 97.59 kDa of the gluten network made with MGF.

**
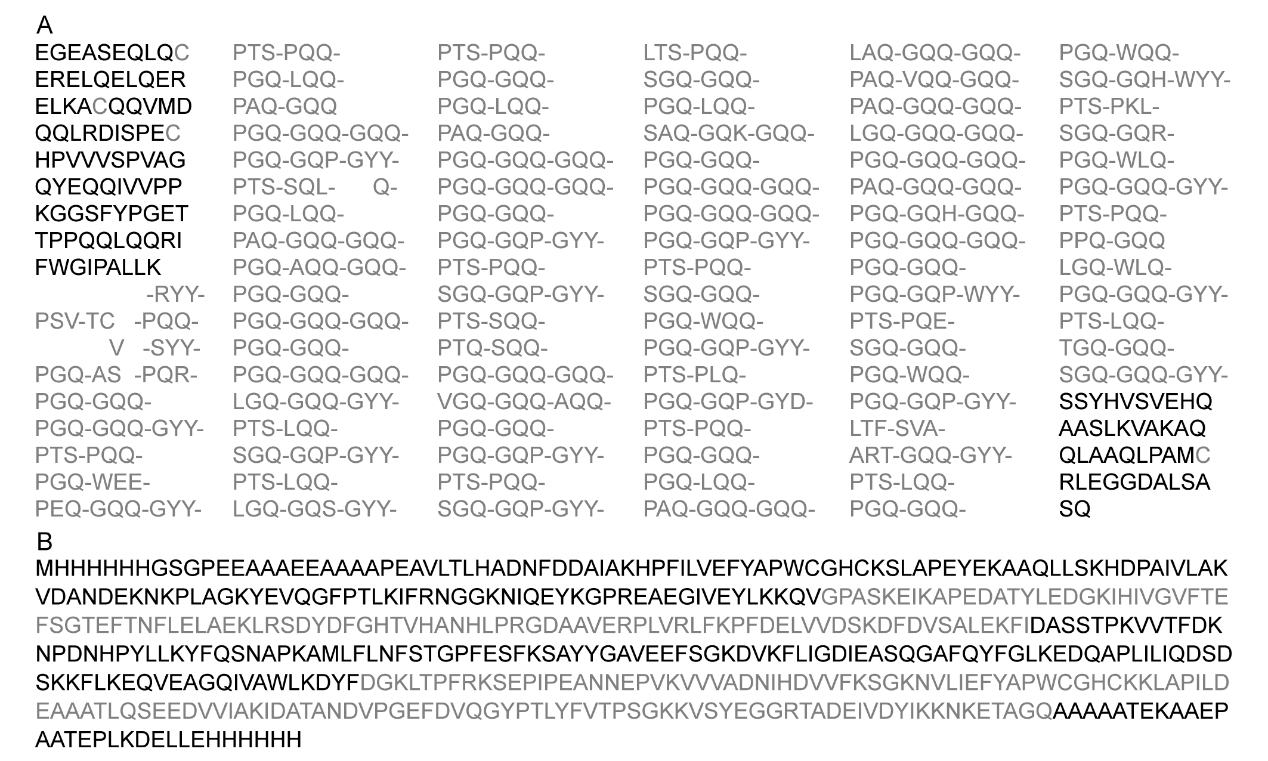
**

**Supplementary Fig. 4| HMW-GS Glutenin 1Dx5 and wPDI sequences used in this study**. **A**, The amino acid sequence of HMW-GS 1Dx5. Black represents the N- and C-terminal domains flanking the central repetitive domain (light colored). Cystine residues in both terminal domains are shown in grey. **B**, The amino acid sequence of wPDI, the different colored regions represent a domain, b domain b’ domain, a’ domain, and the c-tail, respectively.


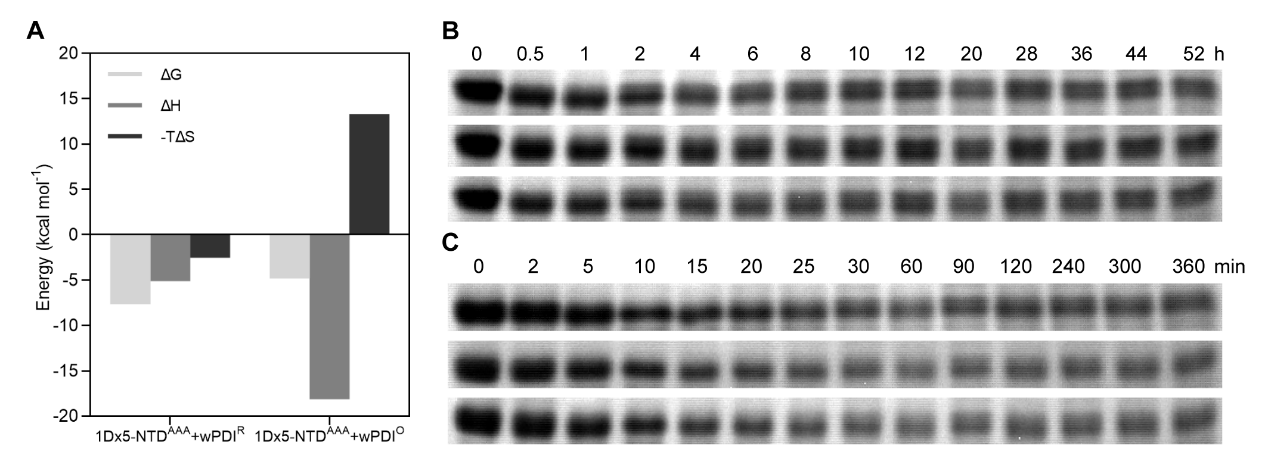


**Supplementary Fig. 5|** **Thermodynamic measurements of the 1Dx5-NTD interaction with wPDI and its crosslinking.** **A,** Enthalpy, calculated Gibbs free energy, and minus temperature times entropy of the 1Dx5-NTD^AAA^ variant interacting with wPDI^R^ and wPDI^O^. **B,** Remaining HMW-GS 1Dx5-NTD protomers after self-crosslinking in the absence of wPDI at different time intervals. **C,** Remaining protomers after HMW-GS 1Dx5-NTD self-crosslinking in the presence of wPDI at different time intervals. Please note the difference in time units.


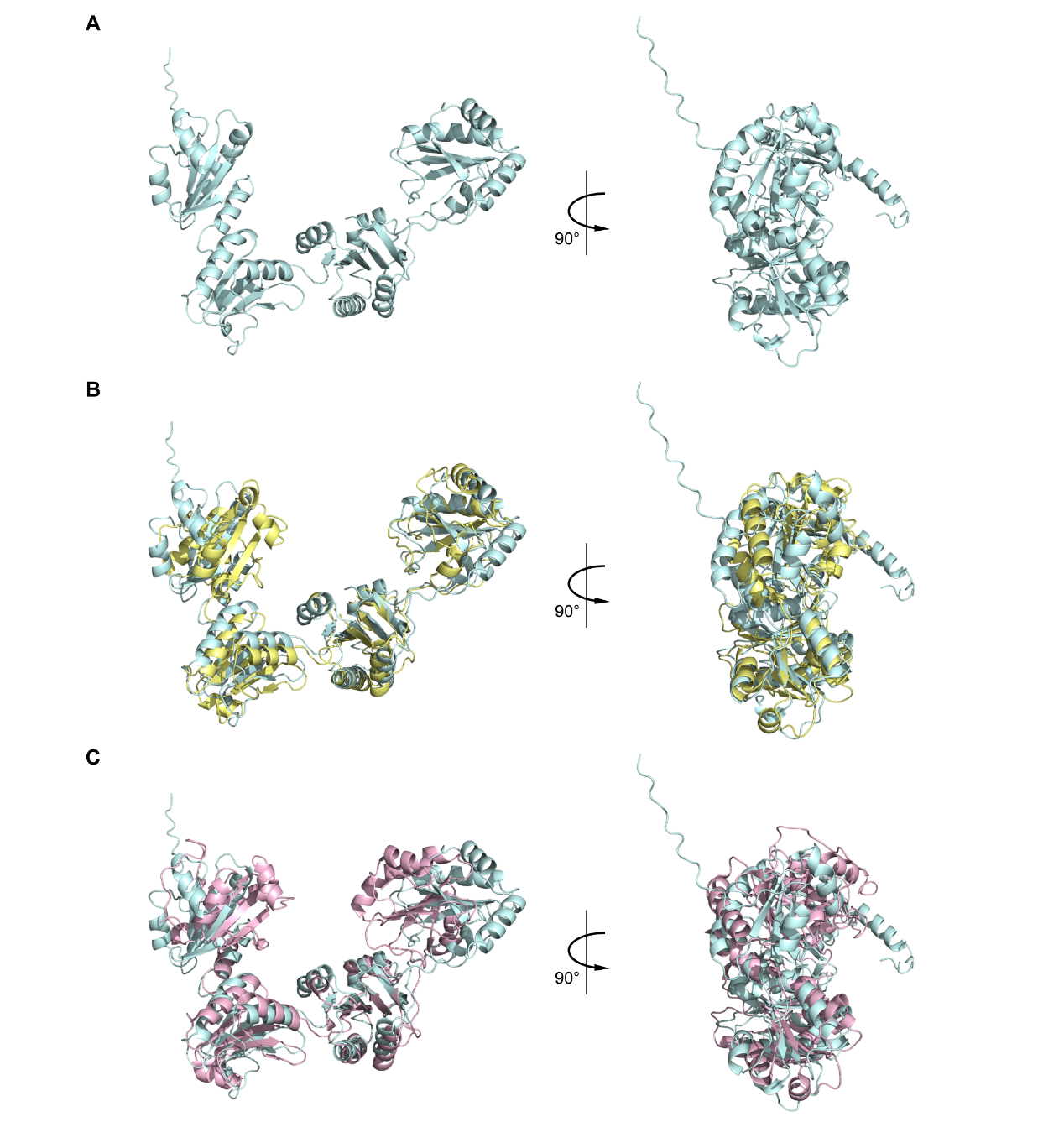


**Supplementary Fig. 6| Predicted wPDI structure model. A,** wPDI structure model predicted with AlphaFold (cartoon presentation in palecyan). **B,** Overlay of the predicted wPDI structure model (palecyan) with the crystal structure of hPDI^O^ (paleyellow, PDB 4EL1). **C,** Overlay of the predicted wPDI structure model (palecyan) with the crystal structure of hPDI^R^ (lightpink, PDB ID 4EKZ).


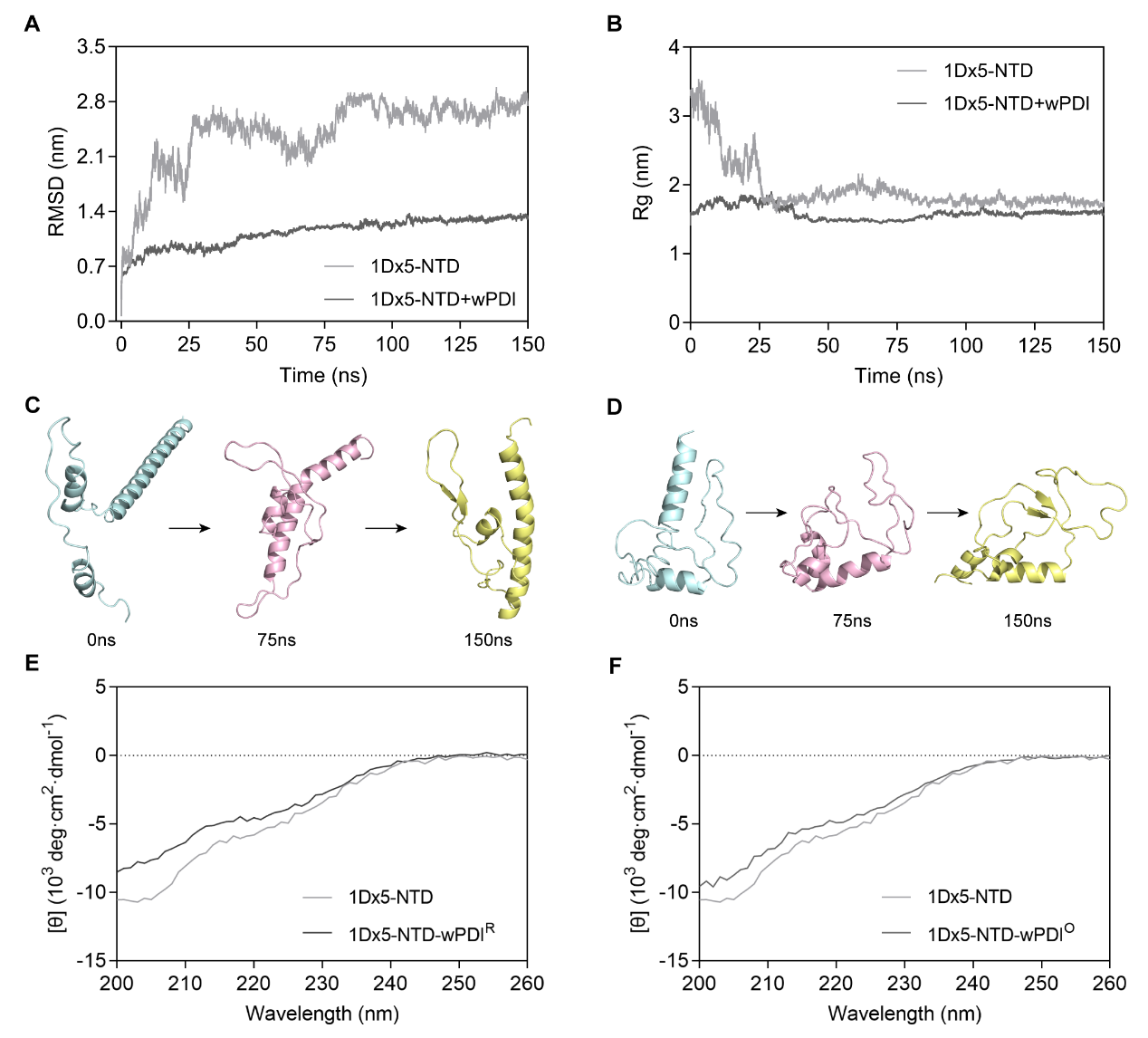


**Supplementary Fig. 7| Molecular dynamics of HMW-GS 1Dx5-NTD and its interaction with wPDI.** **A,** RMSD in the dynamics of HMW-GS 1Dx5-NTD alone and interacting with wPDI. **B,** Rg in the dynamics of HMW-GS 1Dx5-NTD alone and interacting with wPDI. **C,** Molecular conformation of HMW-GS 1Dx5-NTD alone and **D,** interacting with wPDI (wPDI not shown) at different simulation time intervals. **E,** CD spectrum of reductive 1Dx5-NTD in the absence of wPDI and in the presence of wPDI^R^ (with the wPDI^R^ signal deducted). **F,** CD spectrum of oxidized 1Dx5-NTD in the absence of wPDI^O^ and in the presence of wPDI^O^ (with the wPDI^O^ signal deducted).
